# Pneumomediastinum From Vacuum Disc and Vertebral Trauma

**DOI:** 10.7759/cureus.16942

**Published:** 2021-08-06

**Authors:** Mark A Ravago, Pierre D Maldjian

**Affiliations:** 1 Department of Radiology, Rutgers University, Newark, USA

**Keywords:** pneumomediastinum, ct, vacuum disc, trauma, annulus fibrosis

## Abstract

We present a case of pneumomediastinum associated with an endplate fracture adjacent to a vacuum disc. Pneumomediastinum from trauma can be due to damage to the lungs, airway, or esophagus. In this case, we present a unique complication of the vacuum disc phenomenon in which vertebral injury at the site of a vacuum disc releases gas bubbles into the mediastinum. We believe that compressive forces from the trauma produced a disruption of the annulus fibrosis and forced gas previously sequestered in the intervertebral disc space to escape into the mediastinum.

## Introduction

Pneumomediastinum is the abnormal presence of air within the mediastinum. Pneumomediastinum can result from any condition in which air is released into the mediastinum from the lungs, airways, or gastrointestinal tract such as alveolar or esophageal rupture, surgery, and pulmonary barotrauma. Signs and symptoms of pneumomediastinum include chest pain, dyspnea, and subcutaneous emphysema. Serious or life-threatening pneumomediastinum occurs when a significant amount of air causes blood vessel or tracheal obstruction [1]. While traumatic pneumomediastinum can be associated with various mechanisms, to our knowledge, there has not been a reported case of pneumomediastinum caused by the release of air from a vacuum disc. We report a case of pneumomediastinum secondary to a vertebral endplate fracture producing leakage of gas from the adjacent intervertebral disc space. 

## Case presentation

**Figure 1:**
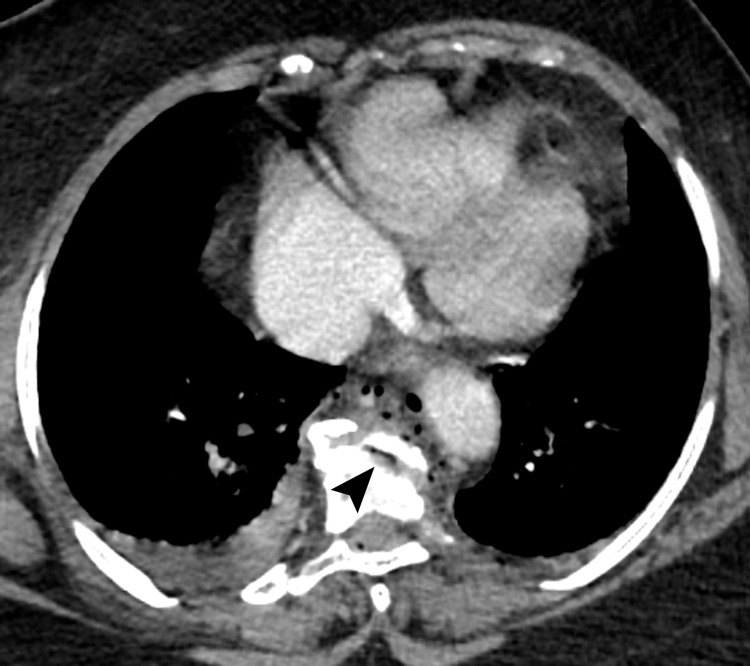
Axial image from CT scan of the chest at the level of the top of the T9 vertebral body shows fracture through the superior endplate containing a small amount of residual air in the disc space (arrowhead). Note multiple small air bubbles in the prevertebral region (pneumomediastinum).

**Figure 2:**
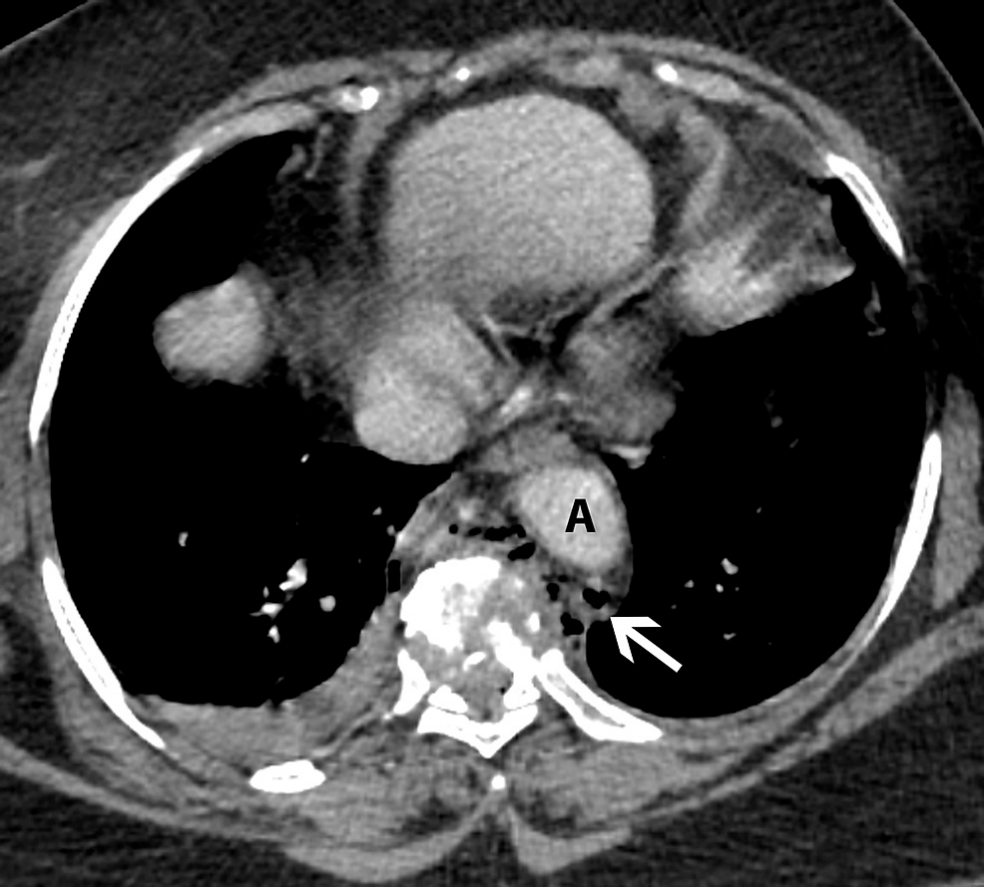
Axial image at a slightly lower level shows fracture of the vertebral body with air bubbles (arrow) between the spine and aorta (A).

**Figure 3:**
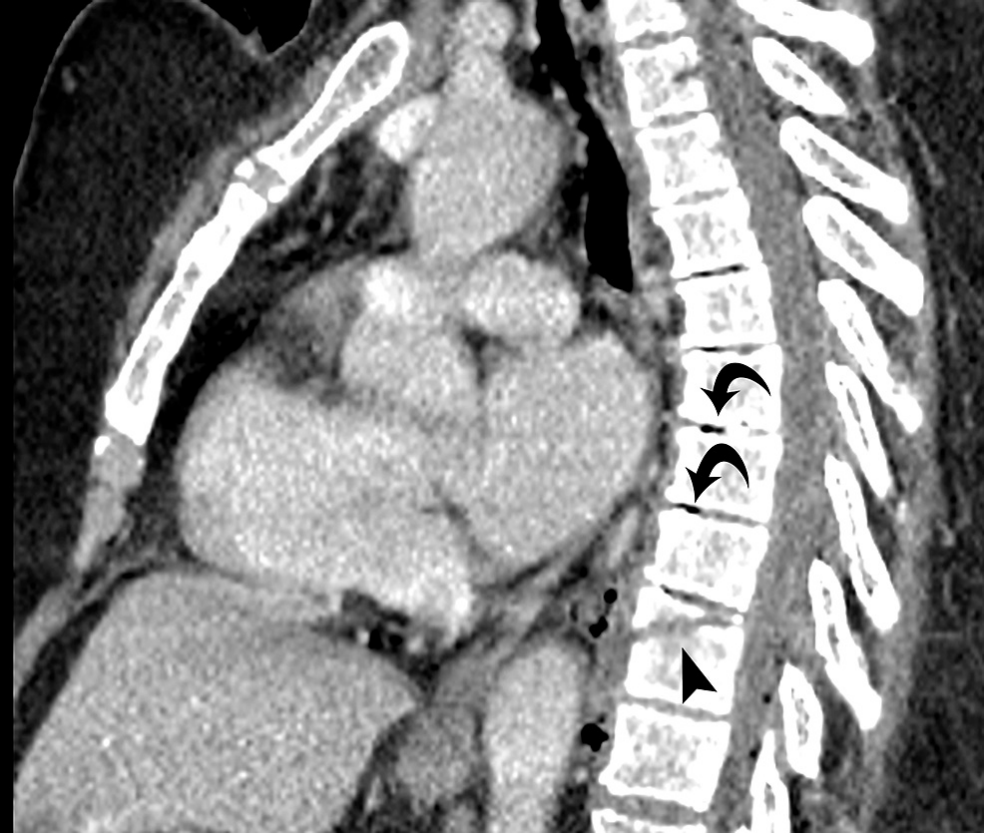
Reformatted sagittal image shows fracture through the vertebral body (arrowhead) with vacuum discs at multiple higher levels (curved arrows).

**Figure 4:**
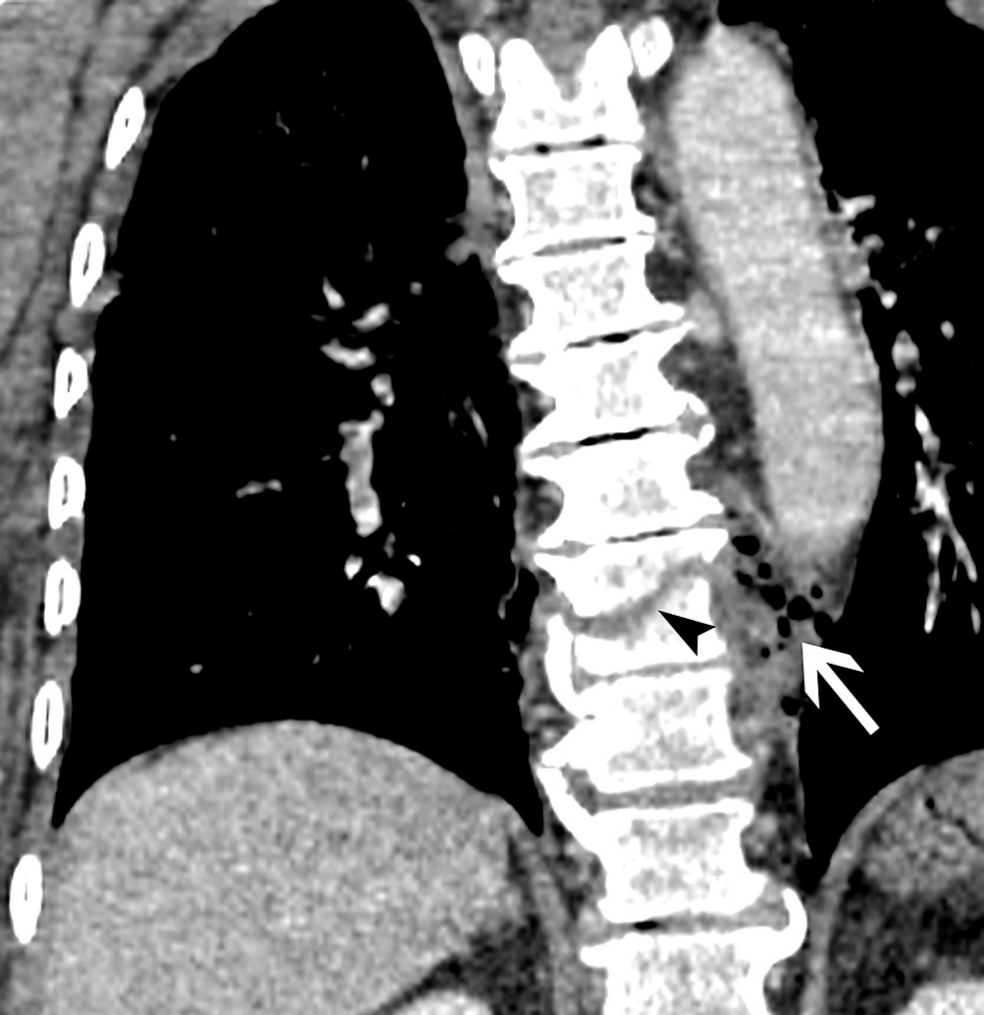
Coronal reformatted view shows fracture through T9 vertebral body (arrowhead) with small air bubbles (arrow) in the left paraspinal region.

**Figure 5:**
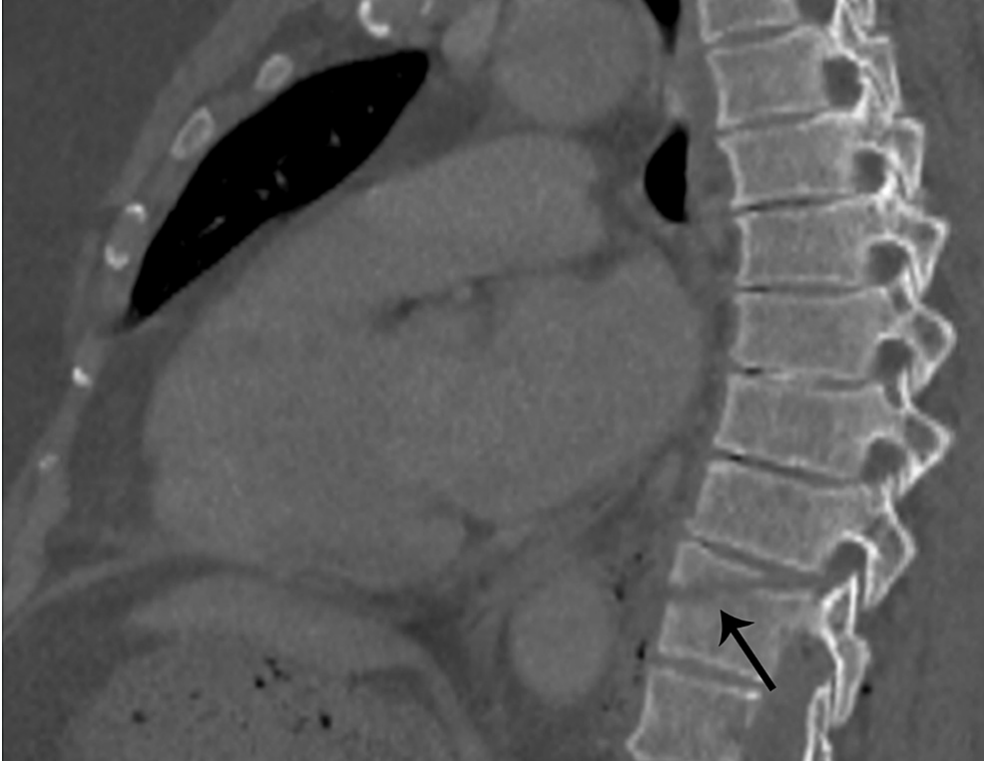
Reformatted sagittal image in bone window shows fracture through the vertebral body (arrow).

**Figure 6:**
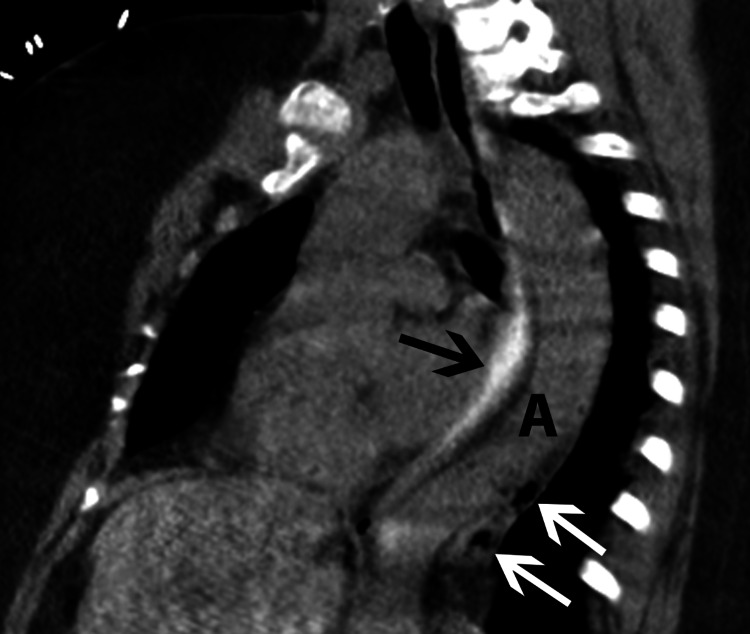
Reformatted oblique sagittal image from subsequent CT esophagram shows contrast in esophagus (black arrow) with no evidence of esophageal leak. Air bubbles are present in the posterior mediastinum (white arrows) posterior to the descending thoracic aorta (A).

A 66-year-old woman with no significant past medical history presented to the emergency department following a motor vehicle accident. The patient was an unrestrained backseat passenger whose vehicle was struck from behind. She complained of severe back pain in the lower thoracic region. The patient had no neurologic deficits. A computed tomography (CT) scan of her chest, performed as part of her trauma evaluation, showed an oblique fracture through the superior endplate and vertebral body of the ninth thoracic vertebra (T9) with small mediastinal air bubbles in the adjacent prevertebral and left paravertebral regions. A small amount of air in the intervertebral disc space was present at T8-T9 along with the vacuum disc phenomenon at multiple higher levels (Figures 1-5). There were no other injuries with no evidence of pneumothorax, lung laceration, or airway laceration. Subsequent CT esophagram showed no evidence of esophageal injury (Figure 6). The pneumomediastinum was attributed to leakage of air from a vacuum disc at T8-T9. The patient was treated with open reduction of the T9 vertebral fracture and spinal fusion from T8-T10. Her postoperative course was uneventful and she was discharged to a rehabilitation facility.

 

## Discussion

The vacuum disc is an accumulation of gas, mainly nitrogen, within the intervertebral disc. It is a common radiologic finding and can be identified in up to 46% of cases on CT [2]. It is believed that extension of the spine produces negative pressure and sucks nitrogen from the surrounding tissues out of solution into the joint space. The gas may then fill clefts within the intervertebral disc produced by disc degeneration [3]. While the formation of vacuum discs is relatively common, leakage of air from discs is rare. There have been cases in which gas from intervertebral discs has seeped into the epidural space or spinal canal, resulting in pneumorrhachis [4-7]. In these cases, it was suspected that the posterior leakage of air occurred due to tears in the annulus fibrosis. 

To our knowledge, there have been no previously reported cases with anterior release of gas from a vacuum disc resulting in pneumomediastinum. A possible explanation for the rarity of this phenomenon is the anatomy of the annulus fibrosis. The anterior margin of the annulus fibrosis is thicker and more resistant to disc herniation and rupture than the posterior border [8]. There are also multiple layers of obliquely oriented connective tissue fibers along the anterior aspect which are more reinforcing than the predominantly vertically oriented fibers along the posterolateral borders [9]. The presence of vacuum discs at multiple levels in our patient indicates susceptibility to degenerative disc disease and weakening of the annulus fibrosis. We suspect that the fracture through the superior endplate produced a disruption of the anterior and anterolateral margins of the annulus fibrosis and the compressive forces from the trauma forced gas previously sequestered in the disc space to escape into the mediastinum. 

## Conclusions

In conclusion, while trauma can cause pneumomediastinum from damage to the lungs, airway, or esophagus, we present an unusual mechanism. Spinal injury at the site of a vacuum disc can also release gas bubbles into the mediastinum. We propose that the compressive forces from trauma can produce disruption of the annulus fibrosis resulting in the release of gas previously sequestered in the intervertebral disc space. This case report is intended to present a unique complication related to the vacuum disc phenomenon and to expand on considerations for causes of posttraumatic pneumomediastinum.

## References

[1] Kouritas VK, Papagiannopoulos K, Lazaridis G (2015). Pneumomediastinum. J Thorac Dis.

[2] Larde D, Mathieu D, Frija J, Gaston A, Vasile N (1982). Spinal vacuum phenomenon: CT diagnosis and significance. J Comput Assist Tomogr.

[3] Yanagawa Y, Ohsaka H, Jitsuiki K (2016). Vacuum phenomenon. Emerg Radiol.

[4] Demierre B, Ramadan A, Hauser H, Reverdin A, Rilliet B, Berney J (1988). Radicular compression due to lumbar intraspinal gas pseudocyst: case report. Neurosurgery.

[5] Kim CH (2007). Pneumorrhachis and paraspinal air with vacuum disc: case report and literature review. J Korean Neurosurg Soc.

[6] LaBan MM, Zaidan S (2011). Vacuum lumbosacral discs leaking nitrogen bubbles into spinal fluid. Am J Phys Med Rehabil.

[7] Yun SM, Suh BS, Park JS (2012). Symptomatic epidural gas-containing cyst from intervertebral vacuum phenomenon. Korean J Spine.

[8] Simon J, McAuliffe M, Shamim F, Vuong N, Tahaei A (2014). Discogenic low back pain. Phys Med Rehabil Clin N Am.

[9] Tenny S, Gillis CC (2020). Annular disc tear. StatPearls [Internet].

